# Synthesizer: Expediting synthesis studies from context-free data with information retrieval techniques

**DOI:** 10.1371/journal.pone.0175860

**Published:** 2017-04-24

**Authors:** Lisa M. Gandy, Jordan Gumm, Benjamin Fertig, Anne Thessen, Michael J. Kennish, Sameer Chavan, Luigi Marchionni, Xiaoxin Xia, Shambhavi Shankrit, Elana J. Fertig

**Affiliations:** 1 Department of Computer Science, Central Michigan University, Mt Pleasant, MI, United States of America; 2 Ronin Institute for Independent Scholarship, Montclair, NJ, United States of America; 3 Department of Marine and Coastal Sciences, Rutgers University, New Brunswick, NJ, United States of America; 4 Colorado Center for Personalized Medicine, University of Colorado Denver, Denver, CO, United States of America; 5 Department of Oncology, Johns Hopkins University, Baltimore, MD, United States of America; Swiss Institute of Bioinformatics, SWITZERLAND

## Abstract

Scientists have unprecedented access to a wide variety of high-quality datasets. These datasets, which are often independently curated, commonly use unstructured spreadsheets to store their data. Standardized annotations are essential to perform synthesis studies across investigators, but are often not used in practice. Therefore, accurately combining records in spreadsheets from differing studies requires tedious and error-prone human curation. These efforts result in a significant time and cost barrier to synthesis research. We propose an information retrieval inspired algorithm, Synthesize, that merges unstructured data automatically based on both column labels and values. Application of the Synthesize algorithm to cancer and ecological datasets had high accuracy (on the order of 85–100%). We further implement Synthesize in an open source web application, Synthesizer (https://github.com/lisagandy/synthesizer). The software accepts input as spreadsheets in comma separated value (CSV) format, visualizes the merged data, and outputs the results as a new spreadsheet. Synthesizer includes an easy to use graphical user interface, which enables the user to finish combining data and obtain perfect accuracy. Future work will allow detection of units to automatically merge continuous data and application of the algorithm to other data formats, including databases.

## Introduction

**Fig 1:**
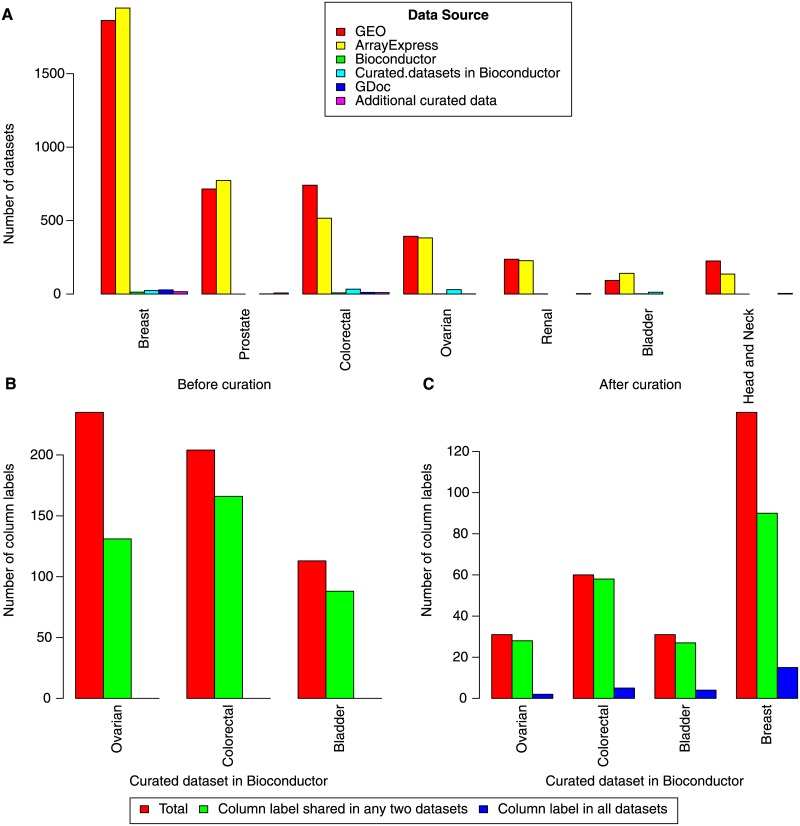
Number of datasets available before and after curation.

Scientists have access to an extensive and varied array of high-quality datasets collected by independent laboratories/studies. The availability of data has resulted in synthesis studies. These synthesis studies combine data across independent studies to arrive at new and exciting conclusions. Many of the independent studies are collected into public domain databases so that they are readily accessible to researchers. For example, in the case of human cancers, databases such as Gene Expression Omnibus (GEO) and Array Express contain high-throughput, multi-platform characterizations of tumors from thousands of independent datasets (see Fig 1a for numbers of datasets per database). In regards to cancer research, combining data in public databases also increases the number of tumors available for molecular profiling by any individual investigative team [1] enabling public domain analysis tools sufficient sample size to validate molecular biomarkers [2, 3].

Though synthesis studies do not involve manual data collection, there are still costs associated with using disparate datasets. A critical and time intensive first step in using multiple datasets is to merge the annotations of each sample manually. These sample annotations are frequently provided as labels in data tables and often do not conform to any standards. For example, in a set of highly utilized genomic databases, none of the column names for sample phenotypes were shared across every dataset preceding curation (Fig 1b). Only 25% of the sample annotations were shared across the datasets after intensive human curation to match sample phenotypes to a common standard (Fig 1c). In regards to cancer data, there are thousands of genomic datasets in the public domain. However, only a handful of the datasets features curated clinical annotations (as illustrated in Fig 1a) due to the time cost associated with manual annotation [4, 5].

In the past, both information retrieval (IR) and natural language processing researchers have worked to map datasets to existing ontologies in order to aid scientists when synthesizing datasets. Numerous systems, including MetaMap [6] and Mgrep [7] have been developed to link biomedical text to existing terms and index biomedical literature. These programs are linguistically sophisticated, employing word sense disambiguation, text negation, and detecting author-defined abbreviations and acronyms. [8] apply MetaMap and Mgrep to obtain ontology based labels of genomics data. This work has also been used to select appropriate datasets to analyze to explore the interaction between phenotype, disease, environmental and experimental data [9] and impute phenotypes [10]. Nonetheless, manual curation is still a critical part of preparing sample annotations to perform the robust statistical analyses required in meta-analysis studies.

In the quest to create more useful queries, the Information Retrieval field is advancing in its ability to recognize commonalities between words. In its infancy, information retrieval systems related words by searching for exact matches in sets of documents. Today’s IR systems are more advanced and can define a “semantic context” that describes related words, in a process called query expansion. These contexts could frequently derive from synonyms, hypernyms, and collocates between words in lexical databases like WordNet [11] or the Corpus of Contemporary American English (COCA) [12]. Diverse applications built on IR concepts such as Google, metaphor identification software [13], and sentiment analysis [14] all use query expansion. We propose that the ability to integrate text data without an explicit context can be used to annotate samples across a wide range of biomedical studies.

To our knowledge, there is currently no publicly accessible software that automatically combines unformatted spreadsheets from disparate datasets using NLP concepts. The closest application in regards to functionality was Google Refine. The Refine software allowed the user to import data in various formats and did automatically combine identically labeled columns. However, unlike the software presented in this paper, Google Refine did not automatically combine data with differently named columns. Also, Google Refine has been renamed to OpenRefine [15], and is no longer supported by Google.

We propose a novel information retrieval algorithm to mine and standardize data tables by introducing semantic context. NLP concepts used in the proposed algorithm, such as term collocation and cosine similarity, are commonly used in text mining applications, however, to our knowledge they have not previously been applied to merge unformatted data. The proposed IR/NLP algorithm, combined with a user-friendly drag and drop web application called Synthesizer facilitates the seamless standardization of data. We demonstrate the efficacy of the algorithm and software to standardize patient phenotype annotation using a comprehensive collection of genomics datasets for four human cancers. Only data from one of the cancer types (Head and Neck Squamous Cell Carcinoma, HNSCC) was used to tune the Synthesize algorithm, with all other datasets used for testing. The algorithm had high accuracy when tested to standardize sample annotations for other cancer types and three ecological datasets, suggesting its general applicability to other disciplines.

## 1 Methods

### 1.1 The synthesize algorithm

**Fig 2:**
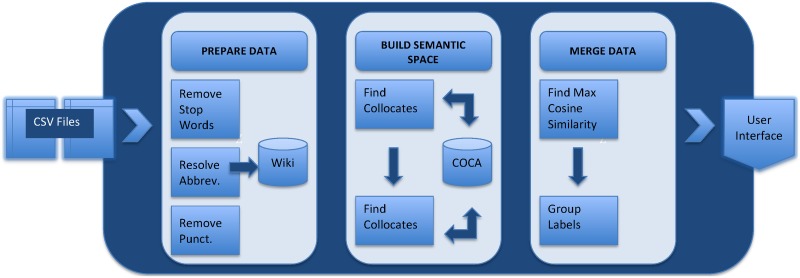
System figure for the synthesizer application.

We developed a novel information retrieval algorithm to standardize sample annotations called Synthesize (workflow outlined in Fig 2). The inputs to the algorithm are files in comma separated value (CSV) format. The algorithm merges labels of sample annotations based on their similarity in a “semantic space”. For explanatory purposes, we will use an ongoing example which includes two spreadsheets, A and B (see Table 1).

**Table 1:** Example spreadsheets with data types.

Spreadsheet	Column header	Column Datatype	Column values
A	gender	discrete	male, female
B	sex	discrete	male, female
B	grade	discrete	moderate, poor, well

To begin merging columns, the algorithm queries the COCA database to find word collocates for each of the terms for header and values in a given column of data. We will define a set for each column of data containing its header string and all individual words (or unigrams) in that column. In the case that either the column header or the value is a phrase containing multiple words, each word in that phrase is added independently to the set. For a given column *x* in spreadsheet *k*, this set is referred to as $l_{x}^{k}$. For example, the set generated for $l_{gender}^{A}$ in Spreadsheet A would be the terms “gender”, “male”, “female”. The set $l_{x}^{k}$ defined from each column is then extended to include the unique set of collocates for all of its terms as ($c_{x}^{k}$). Thus, the set generated for $c_{gender}^{A}$ would include “gender”, “male”, “female” and then collocates of each term such as “difference”, “age”, and all other collocates of gender, female, and male (enumerated in Table 2.) Note that at this stage each term in the set is not stored as a frequency count but is marked as either 1 or 0 to indicate presence or absence.

**Table 2:** COCA collocates for the gender column.

Term	Collocates
gender	difference, age, ethnic, effect, issue, significant, race, role, between, class
female	both, participate, voice, bodily, figure, student, male, than, athlete, first
male	young, black, white

**Fig 3:**
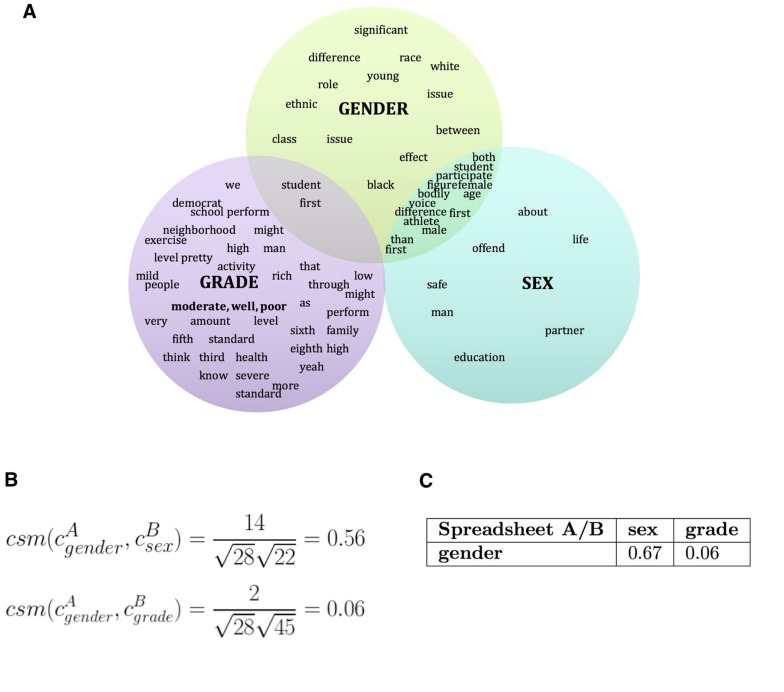
A) Venn diagram showing the common collocates between the column “gender” in spreadsheet A ($c_{gender}^{A}$) and column “sex” in spreadsheet B ($c_{sex}^{B}$) of Table 1, respectively. B) Eq 2 applied to running example to compute the C) matrix of cosine similarity measures between spreadsheet A and B (*cs*_*matrix*).

Now that each set $c_{x}^{k}$ contains the original terms with their collocates, the next step in the algorithm finds the columns from the input spreadsheets to merge (Fig 3A). To begin this process, we calculate the cosine similarity between each set of composite labels in each pair of spreadsheets. Cosine similarity is a formula that leverages the number of features two vectors have in common versus the number of features that they do not have in common. In this case, it is calculated for the sets *c* as follows:

$$\begin{array}{c} \frac{\left|c_{x}^{j}\cap c_{y}^{k}\right|}{\sqrt{\left|c_{x}^{j}\right|}\sqrt{\left|c_{y}^{k}\right|}} \end{array}$$ (1)

where |⋅| refers to the size of the specified set, for all columns *x* in spreadsheet *j* and all columns *y* of spreadsheet *k*. Note that Eq 1 is the Ochiai coefficient, which is essentially identical to the classical equation for cosine similarity when used with bitwise vectors. We provide the application of this metric to our example in Fig 3B. When comparing pairs of spreadsheets, this comparison results in a matrix with dimensions given by the number of columns in each matrix that is analyzed (called *cs*_*matrix*, Fig 3C).

The next step of the Synthesize algorithm is to determine which columns to merge. In this step, the Synthesize algorithm finds the pairs of columns from both spreadsheets with the maximum cosine similarity. Specifically, it finds the element in *cs*_*matrix* with the maximal cosine similarity. The algorithm then associates the corresponding row and column of *cs*_*matrix*. The algorithm deletes the selected row and column of *cs*_*matrix* i and the process continues until no comparisons remain. All the pairs associated rows and columns correspond to two distinct columns from each spreadsheet in the comparison. Selection of pairs for merge begins if the resulting estimate for the cosine similarity is above a fixed threshold. In our analyses, we use a threshold value of 0.5 based on our training data (further discussed in Section 2). Therefore, in our running example, the columns sex and gender from Spreadsheets A and B will be merged. The algorithm follows a similar process when there are three or more spreadsheets to merge. In this case, the algorithm applies the comparison between columns and threshold to all columns in every pair of spreadsheets. Rarely, two pairs of combined columns belonging to three spreadsheets will share a column in common. In this case, merging is transitive. Therefore, any columns below the cosine similarity threshold will be merged if their paired columns are merged in the comparison of another pair of spreadsheets.

Any two columns in a pair of spreadsheets that have a cosine similarity below the threshold value are compared to the merging performed in all other pairs of spreadsheets. If they were merged in a distinct set of spreadsheets, they are also merged for the query pair of columns and spreadsheets. This can result in a scenario where a pair of labels in spreadsheets *A* and *B* may have a cosine similarity below the threshold and the corresponding label from spreadsheet *B* and a new spreadsheet *C* might be above the threshold. In this case we are erring on the side of caution and merging all three columns together.

The final task of the algorithm is to export the distinct spreadsheets as a single, merged spreadsheet with rows containing all elements from each dataset. In the merged dataset, the algorithm gives each new set of joined columns a shared “grouping” label. By default, this is the text of the shortest label of the columns in any spreadsheet. Therefore, in the running example, we would choose the term sex as the group name and the label to merge “gender” in spreadsheet A and “sex” in spreadsheet B. However, the user can modify this algorithm assigned label in the Synthesizer visual software (which the authors discuss in the next section). During the merging process, the algorithm does not change the values of each column. The user can also update these values to a standard set of user defined text elements using the Synthesizer software, without performing manual editing of each entry.

### 1.2 Synthesizer interactive annotation merger software

The Synthesizer system is a web-based application built on top of the Cappuccino Application Development Framework, inspired by Apple’s OS X Cocoa APIs. It provides an abstraction layer from HTML and CSS, allowing developers to create rich user interfaces on the web without spending the time to implement interface elements from scratch. Both the framework itself, as well as Synthesizer web application, are written in the Objective-J programming language. The public access Synthesizer software code is available at https://github.com/lisagandy/synthesizer; this code repository also has a link to the online graphical user interface which we will discuss below.

The Synthesizer system uses a graphical user interface (GUI) that employs point and click technology to upload files, merge labels from sample datasets and download merged datasets in a seamless workflow. This GUI is part of an online system that runs in a web browser.

**Fig 4:**
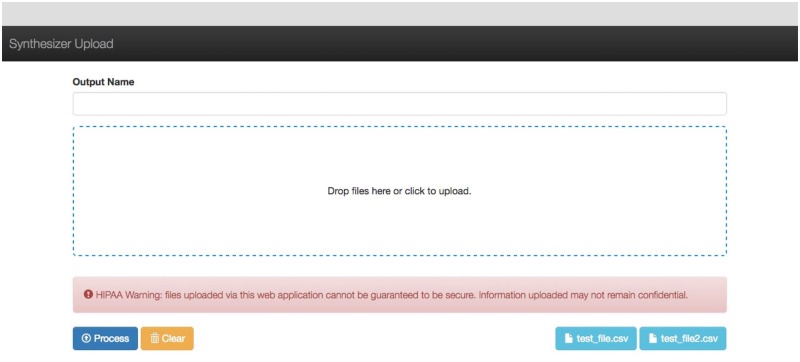
Synthesizer upload files.

The user begins by uploading sample datasets using a simple drag and drop interface (see Fig 4). Alternatively, the user can upload their data by clicking and using the native file browser system for their operating system. The system then uploads the data to a data server and implements the Synthesize algorithm to merge sample annotations.

**Fig 5:**
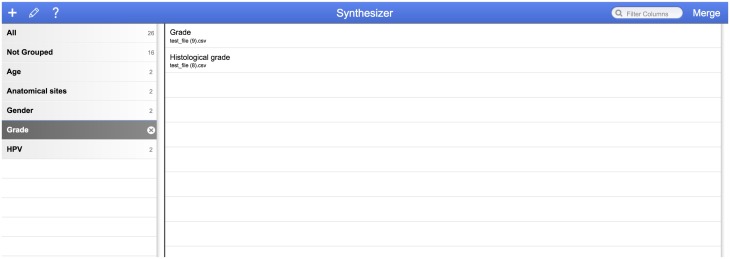
Synthesizer user interface.

As shown below in Fig 5, the Synthesizer interface lists suggested labels on the left-hand side of the screen for merged groups of columns. When the user clicks on the proposed label, the original columns are listed on the right-hand side of the screen.

**Fig 6:**
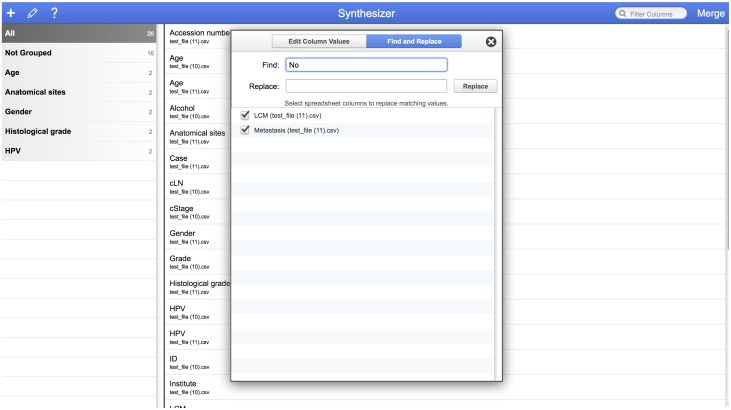
Global find and replace.

The user now has the option to click and move labels between merged groups, and also manually create new groupings. The Synthesizer interface also features the ability to change dataset values individually or by using a global search and replace option (Fig 6).

Once the user has merged labels and changed the data to their satisfaction, they can then obtain a merged spreadsheet by choosing the merge option provided in the interface.

### 1.3 Dataset description

#### 1.3.1 Training data

We trained the Synthesize algorithm on the sample annotations for gene expression from two head and neck squamous cell carcinoma datasets (GSE6791 [16] and GSE3292 [17]) previously curated and used for a synthesis study [18].

#### 1.3.2 Test data

To test the accuracy of the Synthesize algorithm, we collected sample annotations from gene expression datasets measured with Affymetrix hgu133plus2.0 arrays from breast, colorectal, prostate, and renal cancers from Gene Expression Omnibus (accession numbers and references in S1 Dataset). For each cancer type considered, only datasets corresponding to clinical cohorts and with at least ten samples were included. The authors excluded any studies for which no clinical or pathological information was available from the test data. For each dataset, phenotypic information was extracted from the ExpressionSet instance, saved in a text file (TAB-delimited text), and manually curated/reviewed for data consistency and quality. By default, unidentifiable GEO characteristics indices were relabeled based on the information contained in the column itself. However, the individual study of interest defined the relabeling and so relabeling was inconsistent across spreadsheets. Also, no column values were modified. The resulting data files are available in S1 Dataset. The authors converted the datasets to a comma-delimited format (CSV) before being used to train the Synthesize algorithm.

To assess the applicability of the Synthesize algorithm to merge annotations for synthesis studies in scientific disciplines beyond oncology, we tested the algorithm using three Ecology datasets in the public domain (provided in S1 Dataset). The datasets included data from seagrass monitoring, insect abundance, and amphibian life history.

In regards to the seagrass monitoring data, researchers collected samples of eelgrass (Zostera marina L.) and widgeon grass (Ruppia maritima) from Barnegat Bay and Little Egg Harbor Estuary in New Jersey along a gradient of human population density and development. Quadrant, core, and hand sampling of seagrass, following SeagrassNet monitoring and sampling protocols [19] were conducted between 2004 and 2013.

The insect abundance dataset (http://www.pollardbase.org/) is a series of observations of specific lepidopteran taxa made by citizen science programs in North America. Researchers recorded numeric abundance along with a taxon name, location, time, and some environmental measurements. Researchers also collected the data under a moderately standardized protocol, but several significant differences exist in the terms used. The amphibian data set (http://eol.org/, https://github.com/diatomsRcool/MexicanAmphibians) is the result of a literature search for extrinsic and intrinsic traits of Mexican amphibians. Several researchers manually entered data into each spreadsheet.

## 2 Results

### 2.1 Determining the threshold value for merging on test HNSCC datasets

**Fig 7:**
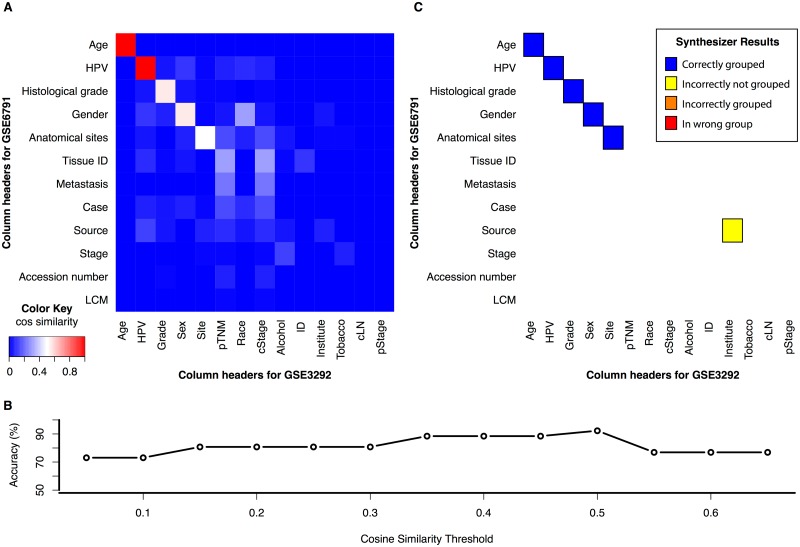
Synthesize cosine similarity threshold selection on two training HNSCC datasets (GSE6791 and GSE3292). A) Cosine similarity between labels and columns of each pair of columns in the two datasets. B) Accuracy of automatic merging as a function of the cosine similarity merging threshold. C) Accuracy of resulting merging for each set of columns using a threshold cosine similarity value of 0.5.

As discussed in Section 1.3, for training purposes, we use the Synthesize algorithm to merge annotations from two sample HNSCC tumor datasets [16, 17]. As part of training, We estimate the cosine similarity between each pair of columns between the two datasets (Fig 7A). Terms with shared column labels have the highest cosine similarity, but the reader can also observe high values for mismatched column labels and values corresponding to the same annotation. The one exception to this trend is the “Institute” and “Source” columns, which both refer to abbreviations referencing the institution collecting the data, and which were not identifiable in the collocate algorithm. High cosine similarity values are also observed for unrelated terms, such as race and gender, but remain smaller than the columns corresponding to the same annotation. The final merging of the Synthesize algorithm depends on the threshold cut-off value between each pair of columns. To determine this value, we compare accuracy as a function of threshold in Fig 7B. As the cosine similarity threshold increases from zero, the accuracy also increases as more columns are incorrectly merged (as they should be left ungrouped). As the cosine similarity surpasses 0.5, accuracy then decreases. Therefore, we select this value of 0.5 as the threshold cosine similarity for merging. With this value, the algorithm combines all columns that should be merged and leaves columns correctly ungrouped (except “Institute” and “Source”) (Fig 7C).

### 2.2 Accuracy of synthesize on merging annotations for cancer genomics datasets

**Fig 8:**
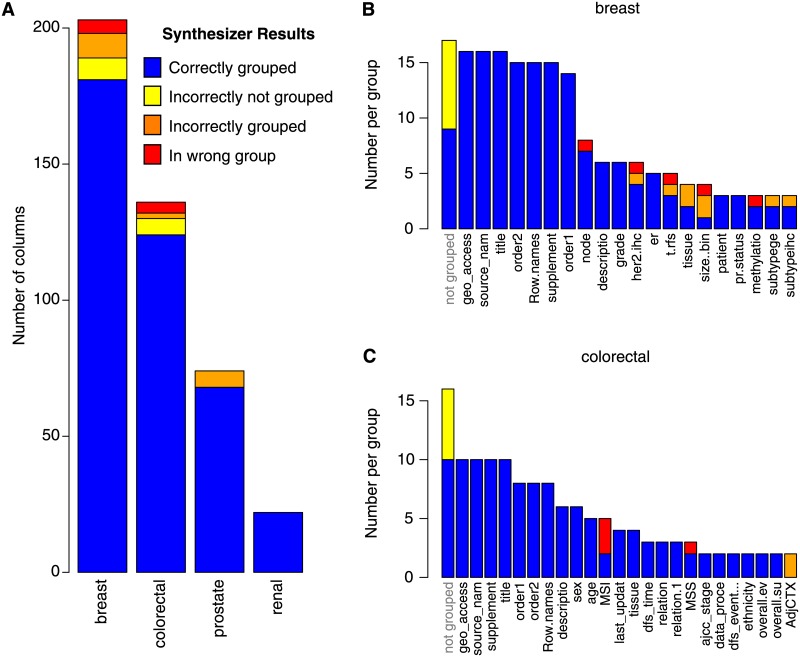
Synthesize accuracy with regards to cancer genomics datasets.

We compare the accuracy of the columns in the cancer datasets combined with Synthesize to hand curation of the annotations. In Fig 8a we give a breakdown of error types. Errors can be broken down into three distinct categories: (1) not grouped by the algorithm but grouped in hand curation (2) grouped by the algorithm but not in hand curation, and (3) placed in different groups by both the algorithm and hand curation. We find that Synthesize accurately merges 89% of 200 columns in 16 spreadsheets for breast cancer, 91% of 137 columns in 10 spreadsheets for colorectal cancer, 92% of 74 columns in 7 spreadsheets for prostate cancer and 100% of the 23 columns in 3 spreadsheets for renal cancer. When examining exact column matches only, the accuracy reduces to 69% of 71 columns for breast cancer, 70% of 43 columns for colorectal cancer, and 53% of 15 columns for prostate cancer and remains at 100% of 5 columns for renal cancer. In the last case, all of the remaining columns are correctly not grouped. This trend indicates that as the number of overall columns increases the accuracy decreases but is still high. The Pearson correlation coefficient between the number of columns and the algorithm accuracy for the four datasets described is −0.87 (p-value of 0.01).

In Fig 8b and 8c we give a further breakdown of the two cancer datasets with the most errors: breast and colorectal. In these datasets, 3% of errors occurred in category 1, 5% in category 2 and 2% in category 3. The most errors occur when columns are grouped together when they should be left ungrouped (category 2) as opposed to being incorrectly ungrouped (category 1) or placed in the wrong group (category 3). Within the breast cancer data, we observe errors merging the column labeled “pr” or “pr.status” in datasets GSE11001, GSE23593 with the “progesterone receptor status” in dataset GSE36774. Also, the system was unable to merge the measurements for the HER2 receptor for a dataset that contained independent measurements with fluorescence in situ hybridization (column labeled “her.fish” in dataset GSE29431) and immunohistochemistry (column labeled “her.ihc” in dataset GSE29431). Finally, the system merged a column labeled “description.1” in the GSE23593 datasets to “methylation.barcode” in the GSE20711 and GSE20712 datasets due to the alphanumeric categories in both.

### 2.3 Accuracy of synthesize on merging annotations for ecological datasets

**Fig 9:**
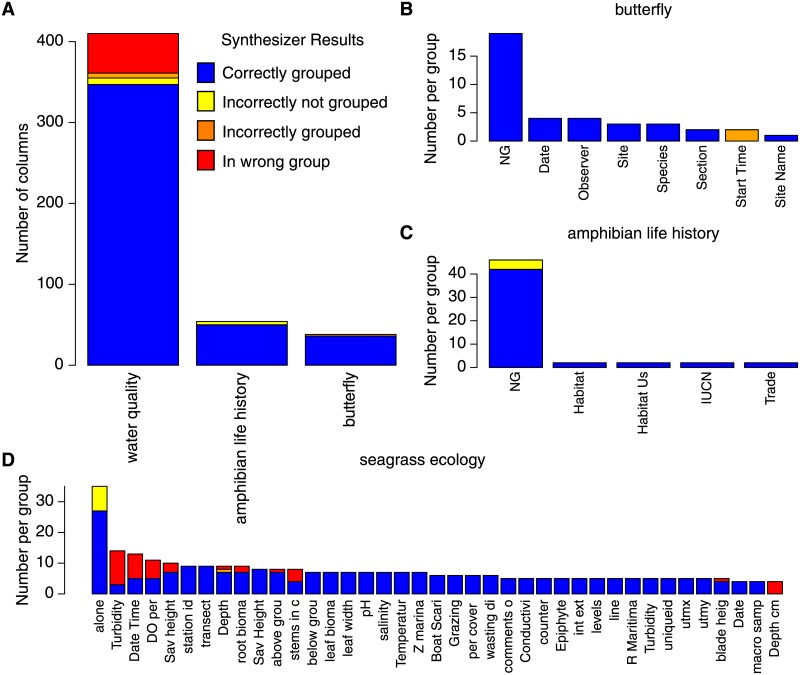
Synthesize accuracy with regards to ecology datasets.

To demonstrate the generality of Synthesizer on unstructured datasets across disciplines, we applied the algorithm to merge several ecological datasets. In regards to this data, the accuracy of the system compared to hand annotation was 85% of 410 columns in 9 spreadsheets correctly merged in regards to seagrass ecology data, 95% of 34 columns for insect data and 92% of 54 columns in regards to amphibian data. As with the cancer data, the accuracy tends towards decreases with the number of columns (Fig 9a, Pearson correlation coefficient of −0.98) that does not reach statistical significance (p-value of 0.11).

When the authors remove exact column matches between spreadsheets, the algorithm accurately merges 77% of 259 columns in regards to seagrass ecology data, 92% of 26 columns in regards to insect data and 92% of 53 columns in regards to amphibian data.

Fig 9b–9d, summarize error types for each ecology dataset. For these data, 2% of the annotations were incorrectly not grouped, 2% grouped when they should be ungrouped, and 10% placed in the wrong group.

## 3 Discussion

This paper presents Synthesizer, which eases the time challenges related to synthesis studies through the ability to quickly and easily combine data. We develop a new NLP algorithm, Synthesize, to merge sample annotations, with an intuitive interface for human-computer interactions to refine merged columns in data. We train the Synthesize algorithm on annotations for head and neck cancer genomics datasets and demonstrate that the algorithm retains high accuracy (ranging from 89%–100%) merging annotations for independent cancer genomics datasets. The overall accuracy for ecology data (ranging from 85%–95%) was comparable to that of the cancer data, despite the fact that the authors trained the system on head and neck cancer annotations from head and neck cancer and abbreviations were resolved against medical terms. In all cases, the number of errors scaled with the total number of columns in the datasets. For example, the seagrass ecology dataset had nearly double the number of columns (410) of the largest cancer dataset (200) and a corresponding decrease in accuracy (85% for seagrass data and 89% for breast cancer). Therefore, we anticipate that Synthesizer would have similar accuracy in merging annotations from new medical and scientific disciplines. The flexible interface for Synthesizer to input unformatted, comma delimited text files of data further supports data merging for such cross-disciplinary synthesis analysis. Errors in merging annotations fall into free common causes: (1) use of abbreviations, (2) multiple columns in one dataset that described a measurement, and (3) bias in the NLP algorithm towards grouping sample labels.

The first factor involves the incorrect resolution of abbreviations by the system. Our system currently uses a generalized list of medical abbreviations which does not only pertain to cancer. We chose a generalized list so that the system could be more adaptable to different types of medical datasets. Still, in regards to cancer datasets, the use of generalized medical abbreviation occasionally causes errors. Consider the case of a column labeled “pr” in the breast cancer data referring to the status of the “progesterone receptor” in a breast tumor. In this instance the system would resolve “pr.status” to “prothrombin ratio status” and therefore would be less likely to merge “pr.status” and a column labeled “progesterone receptor”. The authors plan to mitigate such errors in future extensions of Synthesizer by enabling the user to specify a context-dependent abbreviation dictionary.

The second factor involves the division of clinical information into one of more columns in a dataset. For instance, in the breast cancer dataset, one spreadsheet features a column labeled “her”, referring to the presence of the genomic amplification of the HER2 gene. In another spreadsheet, clinicians record HER2 status with two independent assays by immunohistochemistry (her.ihc) and fluorescence in situ hybridization (her.fish). In this case, a missing metadata issue is present. It is unclear even to a human expert in genomic testing how to combine the two columns without additional information. The current system groups “her” and “her.ihc” together, which is arguably correct, though the algorithm does not group her.fish with the other columns in this case.

Finally, the system is biased towards grouping data, resulting in liberal sample mergers. An example would be a column labeled “description.1” in the breast cancer dataset, which contains information regarding a phenotype encoded using letter and number combinations such as A1, B1, … F1. The system groups this column with “methylation.barcode” from another spreadsheet in the same dataset. Although incorrect, this merge occurred because the methylation barcode column has a series of letters then numbers just like description.1.

While having high accuracy, as with any NLP system Synthesizer may have errors and miss terms in merging datasets. We note that combining data based upon similarity of collocates is general to other pair-wise matching criterion or thresholds. Altering these terms may enhance this accuracy in certain applications. However, it cannot eliminate all NLP errors. Therefore, the human-computer interaction implemented in this software enables users to correct for all errors introduced by the NLP algorithm. We have specifically added features such as the ability to create new groups, move labels between groups to facilitate any remaining manual curation. Also, we have added extra features such as the capacity to change the data in each column or find/replace of data to aid in data modification. As a result, it is possible to obtain perfect accuracy in regards to merging data but with the added benefit of far less work when manually merging all data. Future work is needed to include automatic unit recognition and unit conversion of continuous variables stored in these spreadsheets.

Regarding medical ontologies, we see this work as a complement and not a de facto replacement. One could imagine that in the future Synthesizer could offer a list of widely used ontologies in the medical field and then aid the user in mapping the ontology onto their current dataset. Also, Synthesizer could be used to help researchers map two ontologies onto each other. In future enhancements of the system, this mapping could be saved and used for future merges of datasets. By incorporating medical ontologies, Synthesizer would encourage users to make use of standardized mappings, but with the aid of its automated merge capabilities and easy to use interface.

## Supporting information

S1 Dataset Cancer and ecology datasets. (ZIP)
